# Hypnotic safety suggestions reduce cortisol awakening response and morning heart rate in daily life

**DOI:** 10.1038/s41598-026-52081-x

**Published:** 2026-05-08

**Authors:** Barbara Schmidt, Michael Riede, Martin Walter, Veronika Engert

**Affiliations:** 1 Institute of Psychosocial Medicine, Psychotherapy and Psychooncology, Jena University Hospital, Jena, Germany; 2 Institute of Psychiatry, Jena University Hospital, Jena, Germany; 3 Max Planck Institute for Human Cognitive and Brain Sciences, Leipzig, Germany

**Keywords:** Hypnosis, Suggestion, Cortisol awakening response, Stress, Coping, Long-term effect, Health care, Physiology, Psychology, Psychology

## Abstract

With stress being causally linked to the most frequently occurring mental and physical health problems, effective coping with acute stress is essential for promoting overall well-being and building long-term stress resilience. However, most interventions aimed at improving stress regulation require extended periods of training and practice. In a previous study, we demonstrated that the *Jena Safety Anchor*—a brief, hypnosis-based intervention—effectively reduced subjective stress responses in a controlled laboratory setting using the Trier Social Stress Test (TSST). The present study examined whether this effect extends to everyday life. A total of 80 participants (n = 40 female) were randomly assigned to either a hypnosis group or a control group (n = 40 per group). Over the course of two weeks, all participants collected saliva samples at home to assess their cortisol awakening response (CAR), an indicator of anticipatory stress, reflecting the body’s preparation for expected demands upon waking. Additionally, all participants continuously recorded their heart rate via smartwatches during the two data collection weeks. The hypnosis group received a single guided session of the *Jena Safety Anchor* at the end of the first week, while the control group came to the laboratory but received no intervention. CAR was measured via three saliva samples taken within one hour after waking on specified days. Morning heart rate was measured between 6 and 9 a.m. Results revealed a significant reduction in CAR and morning heart rate in the hypnosis group during the second week, following the intervention. In contrast, no change in CAR and morning heart rate was observed in the control group. These findings show that a single session of the *Jena Safety Anchor* can effectively reduce physiological stress anticipation in the morning. This points to its potential as a rapid, low-effort intervention for enhancing stress coping and fostering resilience. It offers a promising approach to mental health promotion that is both time-efficient and easily integrated into everyday routines.

**Supplementary Information:**

The online version contains supplementary material available at 10.1038/s41598-026-52081-x.

## Introduction

Chronic stress is a major risk factor for a range of physical and mental health conditions, including affective and cardiovascular disorders^1^. Coping effectively with acute stress is a key component of mental well-being^2^. The ability to adapt, recover, or even grow stronger in the face of stress, adversity, or challenging circumstances is known as resilience^3–6^. Resilience is linked to lower stress levels, reduced negative affect, increased positive affect, and fewer physical symptoms^7^. Given rising stress levels and the burden of stress-related illnesses in modern society^8^, there is a pressing need for interventions that enhance stress coping. Stress coping refers to the specific strategies or actions a person uses to manage acute stress. A recent study found that a short, one-week daily audio intervention combining mindfulness and hypnosis reduced subjective stress reactivity more than an active control condition where participants analyzed poems^9^, highlighting the potential of low-effort approaches. Building on this evidence, we developed the Jena Safety Anchor—a post-hypnotic suggestion designed to promote feelings of safety—and tested its effectiveness in a previous study, showing promising reductions in subjective-emotional stress reactivity following an acute psychosocial stress paradigm^10^.

Hypnosis is a focused state of consciousness that enhances responsiveness to suggestion^11^. Typically induced and concluded by a therapist, hypnosis often includes suggestions aimed at altering perception or behavior. When given and tested during hypnosis, these are called *hypnotic suggestions*. Research shows that such suggestions produce specific neural and behavioral effects—for example, imagining a wooden board in front of the eyes reduces brain responses to visual stimuli^12,13^, and imagining wearing earplugs lowers EEG brain responses to sound^14^.

To create a sense of safety, participants are guided to vividly imagine a personal safe place—like a sunny beach or a comforting childhood memory—engaging all senses, such as touch, smell, and sound. This imagined safety reduces brain responses to rewards^15,16^ and helps in medical settings by lowering opioid use^17^ and improving acceptance of treatments such as non-invasive ventilation^18^.

However, when hypnosis and suggestion occur together, it’s hard to separate the effects of the hypnotic state from the suggestion itself. Post-hypnotic suggestions address this by allowing the effect of the suggestion to be reactivated outside hypnosis. We developed the *Jena Safety Anchor*, where participants, while hypnotized, wrote the letter "S" for “safety” on a piece of paper. Later, simply seeing or touching this paper reliably reactivated the feeling of safety—an effect that lasted for weeks^19^.

In a previous study, we demonstrated that a post-hypnotic suggestion of safety, delivered during a hypnosis session before an acute standardized laboratory stressor, significantly reduced subjective stress^10^. Sixty participants received hypnosis with the Jena Safety Anchor. Half were instructed to actively use this anchor during the Trier Social Stress Test (TSST;^20^). Results showed that those who engaged the anchor experienced about 50% less subjective stress both immediately after the TSST and throughout a 90-min recovery period. Notably, none of the participants, regardless of group allocation, exhibited what is conventionally defined as a physiologically significant cortisol response to the TSST (> / = 1.5 nmol/L above baseline levels;^21^). We attributed this to the hypnosis session taking place immediately prior to the stress testing, suggesting a possible carry-over effect of hypnosis-induced relaxation.

To disentangle hypnosis from non-hypnosis effects, the current study randomly assigned participants to a hypnosis group (hypnosis plus Jena Safety Anchor) or a control group (no hypnosis, no anchor), with the hypnosis session scheduled on a day separate from stress testing. To evaluate the real-world applicability of the safety anchor, participants received smartwatches and at-home salivary sampling kits to track stress biomarkers in daily life. In this manuscript, we focus on the cortisol awakening response (CAR) and morning heart rate as indicators of physiological stress regulation. We test whether the Jena Safety Anchor influences stress anticipation in the morning, thereby clarifying its potential as an everyday stress management tool.

The Cortisol Awakening Response (CAR) is a reliable physiological marker that reflects how the body prepares for the upcoming day. It involves a natural rise in cortisol levels within the first 30 to 45 min after waking, driven by the hypothalamic–pituitary–adrenal (HPA) axis. As cortisol plays a central role in managing energy, immune response, and stress regulation, CAR provides valuable insight into how the body anticipates and copes with daily demands^22^.

Importantly, the cortisol awakening response (CAR) is highly sensitive to psychological stress, but it does not represent a response to an acute stressor. In many studies, altered CAR (either elevated or blunted) has been linked to chronic stress, burnout, anxiety, and psychopathology—in other words, dysregulation in CAR often reflects an overtaxed or maladaptive HPA axis^23^. In contrast, lower or more regulated CAR is sometimes considered an indicator of balanced HPA axis functioning and adaptive stress regulation. For instance, reductions in CAR have been observed following compassion-based mental training^24^. Because it is easy to measure and closely tied to mental and physical health, CAR is widely used in stress research. Interventions that can lower or stabilize CAR—such as relaxation techniques, mindfulness, or hypnosis—are seen as promising tools for improving stress resilience^24^.

To index physiological arousal upon waking, morning HR should be measured immediately after awakening and before getting out of bed or moving, as posture and movement strongly influence HR^25^. This period typically reflects the body’s transition from sleep to wakefulness, during which the autonomic nervous system becomes more active^26^. Elevated heart rate during this window, especially when recorded consistently using smartwatches in everyday settings, may indicate heightened sympathetic activity—commonly linked to stress, anxiety, or anticipation of daily demands^27^. Conversely, a lower or stable heart rate may suggest a more relaxed or well-regulated state^28^. Heart rate is jointly regulated by parasympathetic and sympathetic nervous system activity; therefore, increases in heart rate cannot be interpreted as reflecting heightened sympathetic activation alone, as they may result from reduced parasympathetic activity, increased sympathetic activity, or a combination of both^29^.

Smartwatches offer a practical and non-invasive way to capture heart rate data in real-life conditions, providing insights into how an individual’s body responds to waking. Unlike lab-based measures, these devices allow for long-term tracking and assessment of trends, accounting for variations due to sleep quality, physical activity, or psychological stress^27^. Morning heart rate can correlate with cortisol levels and subjective stress reports, reinforcing its role as a proxy for arousal^30^.

Monitoring morning heart rate can be especially useful for understanding patterns related to mental health, recovery, and daily readiness^31,32^. It can help identify chronic stress or overtraining in athletes^33^ and may serve as an early signal of dysregulation in individuals with anxiety or mood disorders^34^.

The present manuscript focuses on the everyday effects of the Jena Safety Anchor using objective physiological measures in participants’ natural environments. Specifically, participants monitored their heart rate using smartwatches and collected saliva samples to assess the cortisol awakening response (CAR) for one week before and one week after the hypnosis intervention. A control group, which did not receive hypnosis, followed the same protocol. We expect that both CAR and morning heart rate would decrease in the hypnosis group following the intervention, reflecting reduced physiological stress. In contrast, we expect no significant changes in the control group.

## Materials and methods

### Participants

A power analysis was conducted using G*power to determine the adequate sample size for our study. We based this power analysis on our main hypothesis, which is the improvement of stress responses from the pre-intervention week to the post-intervention week in the hypnosis group. Meta-analyses on the effect of hypnotic suggestions on mental distress estimate a medium effect of *d* = 0.5^35^. With a power level of 0.9 and an alpha level of 0.05, 36 participants are required in the hypnosis group according to G*power^36^. To account for potential participant drop-out, we included 40 participants in each group, resulting in a total of 80 participants. Participants were recruited via mailing lists, postings on social media and in lectures the Medical Faculty of Jena University. A computer-based randomization tool developed at the Jena University Hospital named Parandies was utilized to allocate participants to their respective groups. In each group, there were 20 female and 20 male participants.

The mean age of participants was 28.2 years (SD = 9.1 years, range 18–58 years) with no significant difference between groups (*p* = 0.95). Participants were predominantly white Europeans. As recommended for stress studies based on cortisol measurements^37^, females had to have a natural menstrual cycle to be included. In consequence, participating females did not take any form of hormonal birth control, did not breastfeed and were not peri- or menopausal. General inclusion criteria were that participants are at least 18 years old, do not smoke more than 5 cigarettes per day, do not drink alcohol more than the low-risk dose (U.S. Department of Health and Human Services & U.S. Department of Agriculture, 2020), do not consume other drugs such as cannabis, do not consume steroid-based medication, do not have acute psychological or neurological problems and do not suffer from cardiovascular disease^38^. The local ethics committee at the Jena University Hospital approved the study (2022-2557_3-BO). Informed consent was obtained from all participants. This study was conducted in accordance with the principles of the Declaration of Helsinki. All methods were performed in accordance with the relevant guidelines and regulations. Participants received 90 euros for participation. The study was preregistered at DRKS, https://www.drks.de/search/de/trial/DRKS00031737/details on 21/04/2023.

### Procedure

After participants were screened on the phone for study inclusion, they completed online questionnaires to measure their trait anxiety via the State Trait Anxiety Inventory, (STAI-T;^39^), chronic stress via the Perceived Stress Scale (PSS-10;^40^) and the Trier Inventory for Chronic Stress (TICS;^41^), sleep quality via the Pittsburgh Sleep Quality Index (PSQI;^42^), general health via the Patient Health Questionnaire (PHQ;^43^), emotion regulation strategies via the Heidelberg Form for Emotion Regulation Strategies (H-FERST;^44^), and depression via the Beck Depression Inventory (BDI;^45^).

Then, an appointment was made to hand out the Withings ScanWatch 2 smartwatches and salivettes for home saliva collection. For the cortisol awakening response, according to current recommendations^46,47^, participants had to collect one saliva sample immediately after awakening as well as 30 min and 45 min later on two days in the first experimental week. In line with established recommendations, we ensured that the two assessment days were non-consecutive weekdays with at least one day in between (e.g., Monday and Wednesday or Tuesday and Thursday). Participants were explicitly instructed to select two non-consecutive working days and to maintain their regular daily routines. They were asked not to modify their sleep behavior or morning routines during the assessment period. They were explicitly asked to avoid brushing their teeth, drinking coffee, tea, or juice, and eating immediately before sampling. In addition, they were instructed to refrain from food intake, beverages other than water, smoking, or exercise until completion of the sampling procedure. Participants documented awakening time and relevant contextual factors. Light exposure at awakening and other acute state variables were not experimentally manipulated but were minimized through standardized instructions and by restricting assessments to regular weekdays. Further saliva samples as well as participants’ subjective ratings of stress and wellbeing were collected throughout the remainder of the day; these data will be reported elsewhere. To control whether samples were taken on time, pictures with timestamps as recommended by Stalder et al.^47^ were collected. Participants stored the collected saliva samples in their home freezers.

After the first week, participants returned to the laboratory to hand in their salivettes and receive the second home sampling batch. Participants from the experimental group also took part in the hypnosis session. Data collection happened as in week 1. Participants returned to the lab for one more visit to hand over the second batch of collected saliva samples and return all used equipment. In this final experimental session, we also performed a TSST with physiological and psychological measurements. This data, as well, will be reported elsewhere. In the end of the study, we asked participants in the hypnosis group if they used the Jena Safety Anchor in their everyday life after the hypnosis session (yes, no, no answer).

### Hypnosis session

The participants in the hypnosis group received their hypnosis session between the two experimental weeks. Hypnosis was provided by trained medical students, supervised by the first author of the study. The participant and the hypnotist sat down in comfortable chairs and the hypnotist started the hypnosis session with a hypnosis introduction following the Stanford Hypnotic Susceptibility Scale (SHSS;^48^). To test if participants followed the hypnotic suggestions, the hypnotist used the first item of the SHSS, suggesting that there is a heavy weight in the participant’s right hand. If, in response to this suggestion, the hand sinks downwards, the participant passed the test. Subsequently, the hypnotic suggestion of safety started. The hypnotist guided the participant to imagine that he or she is at a place where he or she feels comfortable and safe. Once this safe place was fully experienced, the participant was instructed to open their eyes and write the letter S for safety on a piece of paper. The hypnotist suggested that every time the participant sees this paper with the letter S on it, folds it and puts it in the pocket, he or she would re-experience the current feeling of safety. Then, the hypnotist ended the hypnotic state. The duration of the hypnosis intervention was about 30 min. Please see our Zenodo repository (10.5281/zenodo.10561059) for the complete wording of the hypnosis intervention. Participants then indicated how safe they feel on a scale from 1 (no change) to 5 (very safe). They also completed the Inventory Scale of Hypnotic Depth (ISHD;^49^) containing 36 items to measure their trance depth. Participants rated each item on a scale from 1 to 4, resulting in a maximal score of 144 for trance depth.

### Statistical analysis

All statistical analyses were conducted using *R* (Version 4.4.1; R Core Team, 2025). Data were cleaned and prepared for analysis prior to statistical testing. Analysis of variance (ANOVA) was performed to examine group differences, followed by post hoc *t*-tests where appropriate to identify specific pairwise differences. Statistical significance was set at *p* < 0.05 for all tests. Data are available via https://zenodo.org/records/17487301.

## Results

### Group differences

The two experimental groups did not differ in any of the pre-study scales (p > 0.1), that is there were no groups differences in terms of state and trait anxiety (STAI-T;^39^, perceived stress (PSS-10;^40^), chronic stress (TICS;^41^), sleep quality (PSQI;^42^), general health (PHQ;^43^), emotion regulation strategies (H-FERST;^44^), and depression (BDI;^45^).

### Lower cortisol awakening response (CAR) with Jena Safety Anchor

Participants collected their CAR saliva samples on two days in the week before and on two days in the week after the hypnosis intervention. We computed the CAR as a difference score between the saliva sample 45 min after waking up (S3) and the saliva sample immediately after waking up (S1). In this analysis, we had to exclude one participant in the control group due to cortisol levels over 100 nmol/l and one participant in the hypnosis group due to eight missing or invalid saliva samples. An ANOVA on the CAR with between factors *group* (hypnosis, control) and within factor *time* (before hypnosis, after hypnosis) revealed a significant main effect of group, *F*(1,372) = 5.03, *p* = 0.03, and a significant interaction of group and time, *F*(1,222) = 4.63, *p* = 0.03. All other effects did not reach significance (*p* > 0.05). Post-hoc *t*-tests show that groups differed significantly before the hypnosis session in the first week, *t*(142) = 3.75, *p* < 0.001 with higher CAR in the hypnosis group (mean = 5.9 nmol/l) than in the control group (mean = 2.0 nmol/l). After the hypnosis session in the second week, the difference in CAR between groups was no longer significant (*p* = 0.7). The effect size of CAR reduction in the hypnosis group, measured as the difference between the week before and the week after the hypnosis session, was Cohen’s *d* = 0.3 indicating a middle-sized effect.

**Fig. 1:**
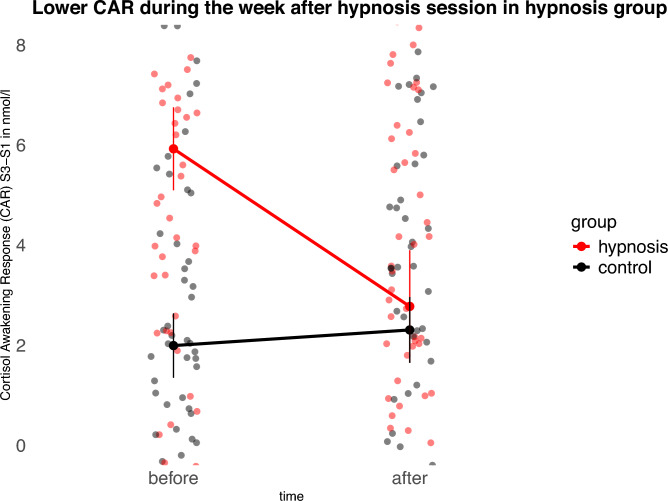
Participants in the hypnosis group showed a significantly reduced cortisol awakening response (CAR) after receiving the Jena Safety Anchor in the hypnosis session compared to participants in the control group. For transparency, a version of this figure displaying the full observed data range is provided in the Supplementary Material.

After the study, participants were asked whether they had used the Jena Safety Anchor in their everyday life. Of the 40 participants in the hypnosis group, 16 reported using it, 16 reported not using it, and 8 did not respond to this question. The association between use of the safety anchor and change in the cortisol awakening response in the hypnosis group was examined using Pearson correlation. There was no significant correlation between anchor use and change in CAR, *r*(30) = 0.02, *p* = 0.91. The 95 percent confidence interval ranged from − 0.33 to 0.37, indicating that the data do not support a meaningful relationship between anchor use and changes in cortisol awakening response (Fig. 1).

### Lower morning heart rate with Jena Safety Anchor

Participants’ heart rate was continuously recorded using a Withings ScanWatch 2 smartwatch for one week before and one week after the hypnosis intervention. To focus on heart rate in the context of the cortisol awakening response, analyses were restricted to data collected in the morning between 6:00 and 9:00 a.m. Heart rate data were averaged into 10-min bins, resulting in 18 time bins across the three-hour morning assessment on each measurement day. Morning heart rate was analyzed using a linear mixed effects model with random intercepts for participants. Fixed effects included *group* (hypnosis, control), *intervention phase* (before, after), their interaction, and *time bin* (1–18) to account for the morning trajectory.

**Fig. 2:**
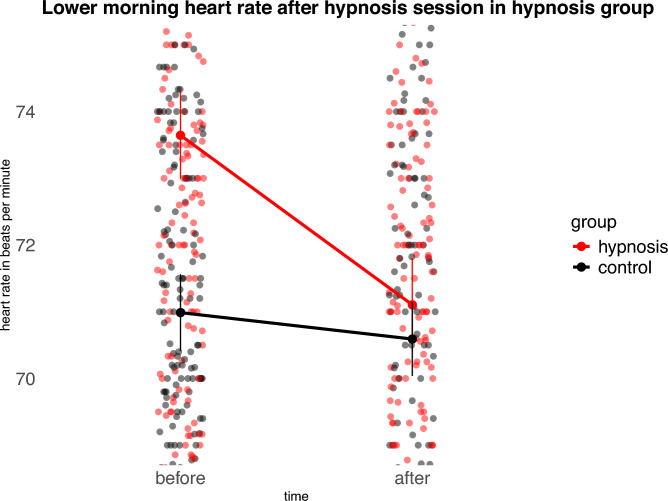
Participants in the hypnosis group showed a significantly reduced morning heart rate after receiving the Jena Safety Anchor in the hypnosis session compared to before. For transparency, a version of this figure displaying the full observed data range is provided in the Supplementary Material.

There was no significant main effect of group. A significant main effect of intervention phase indicated an overall change in heart rate from before to after the intervention. Importantly, the group and intervention interaction was significant, *F*(1, 35,592) = 146.65, p < 0.001. The estimated interaction coefficient (− 1.42 bpm) indicates that the reduction in morning heart rate from before to after the intervention was 1.42 beats per minute greater in the hypnosis group compared to the control group (Fig. 2).

### Hypnotic depth and immediate effect of Jena Safety Anchor in hypnosis session

As an indicator of how deep their hypnotic trance was, participants filled in the ISHD^49^. The mean ISHD score was 95.22 (SD = 16.88), indicating a deep trance state according to Riegel et al.^49^.

Participants rated how safe they felt on a scale from 1 (no change) to 5 (very safe) immediately after the hypnosis session. The mean safety rating after the hypnosis session was 4.5 (SD = 0.8) indicating a strong feeling of safety. The deeper participants’ hypnotic depth measured with the ISHD, the safer participants felt after the hypnosis session, *r* = 0.50, *p* = 0.001.

## Discussion

In this study, we tested whether one hypnosis session is sufficient to affect different indicators of stress regulation in everyday life. During hypnosis, we installed the Jena Safety Anchor. Participants wrote the letter S for safety on a piece of paper, and the hypnotist suggested that every time they look at this paper, they will feel safe again. The control group did not receive a hypnosis session or a safety anchor. To measure everyday stress regulation, participants collected their saliva samples and heart rate during two days of one week before and during two days of one week after the hypnosis intervention. Our data show a significant effect of the hypnosis intervention on cortisol awakening response, indicating that participants in the hypnosis group exhibited a significant reduction in cortisol levels during the week following the intervention compared with the control group. For morning heart rate, the overall ANOVA did not show significant effects; however, exploratory analyses suggested a decrease in morning heart rate in the hypnosis group after the intervention, whereas the control group showed no comparable change.

Furthermore, we observed significant baseline differences between groups in cortisol awakening response (CAR) and morning heart rate, with the hypnosis group showing higher values during the first week. Given that group allocation was performed using a computer-based randomization tool, these differences are likely attributable to chance. It is also possible that the reduction in stress responses in the hypnosis group was more easily achieved due to their initially elevated levels. Importantly, no significant baseline group differences were found for anxiety, stress, depression, or any of the other self-report measures collected prior to the intervention.

The results of this study extends previous research that the Jena Safety Anchor produces robust and long-term subjective feelings of safety^10,19,50^. In the present study, we showed that the Jena Safety Anchor is effective in everyday life. These findings indicate that our hypnotherapeutic technique may improve feelings of stress in anticipation of the upcoming day. If such anticipatory stress can be reduced long-term, a hypnotic safety anchor may help promote mental health and establish resilience.

Reducing the cortisol awakening response (CAR) and morning heart rate may be linked to beneficial changes in participants’ overall physical and psychological health. CAR is a natural rise in cortisol shortly after waking, reflecting hypothalamic–pituitary–adrenal (HPA) axis activity. While a typical CAR supports adaptation to daily demands, both abnormally high and abnormally low CAR have been associated with adverse health outcomes, including stress-related and affective disorders. This inconsistency highlights the importance of interpreting changes in CAR within the study context. In our case, the reduction in CAR is unlikely to indicate heightened stress; rather, it may reflect more efficient stress regulation and resilience, in line with previous interpretations^22,24^.

Similarly, morning heart rate reflects the state of the autonomic nervous system, particularly the balance between sympathetic (fight or flight) and parasympathetic (rest and digest) activity^25^. A consistently elevated morning HR can be a sign of ongoing physiological arousal or poor recovery during sleep, often linked to stress or overtraining^33^. Reducing morning HR may signal enhanced cardiovascular health, improved autonomic balance, and greater recovery capacity^28^.

Together, decreases in CAR and morning HR suggest a more relaxed, well-regulated physiological state. Interventions that lead to such reductions—like relaxation techniques, hypnosis, or mindfulness—may support better stress management, emotional well-being, and thus lead to long-term positive health outcomes for participants.

### Limitations

One limitation of the present study is that the design could not be fully blinded. Because the hypnosis session cannot be meaningfully disguised, participants were aware of whether they received the hypnosis intervention or were part of the control group. This awareness may have introduced demand characteristics or expectancy effects that could have influenced the observed responses. In addition, participants in the hypnosis group spent more time interacting with the experimenters due to the hypnosis session, whereas the control group did not receive a comparable intervention. Therefore, nonspecific factors such as attention or social interaction cannot be entirely ruled out as contributing to the observed effects.

An important limitation concerns compliance with the cortisol awakening response (CAR) sampling protocol. Although participants were instructed to collect samples immediately upon awakening and adherence was monitored (e.g., via time documentation), no strict exclusion criteria for delayed samples (e.g., > 5 min, as recommended in current guidelines) were applied, and compliance information was not incorporated into additional sensitivity analyses. This decision was made to preserve the ecological validity of the everyday life design and to avoid potential bias introduced by selective data exclusion, as noncompliance in naturalistic settings is often systematically rather than randomly distributed.

However, it cannot be ruled out that timing deviations, particularly delays in the first sample, may have influenced the estimation of the CAR. Thus, the present findings should be interpreted with caution. At the same time, not integrating compliance information analytically represents a limitation and a missed opportunity to further strengthen methodological rigor. Future studies may benefit from combining naturalistic designs with objective compliance monitoring and more formal integration of compliance indicators into statistical analyses.

## Conclusion and clinical use

Building on previous findings showing that the Jena Safety Anchor effectively reduces subjective stress in acute social stress situations, the current results demonstrate that its benefits extend to everyday life. Specifically, we found that use of the Jena Safety Anchor was associated with significant reductions in both the cortisol awakening response (CAR) and morning heart rate (HR). This suggests that the intervention not only helps in managing acute, high-pressure scenarios but may also promote a more balanced and resilient stress regulation, particularly less anticipatory stress at the beginning of the day, in daily routines.

CAR reflects the activity of the hypothalamic–pituitary–adrenal (HPA) axis and is sensitive to chronic stress levels^22^. Elevated CAR has been linked to psychological strain, anxiety, and impaired well-being. Similarly, a heightened morning HR can be a sign of autonomic dysregulation and an indicator of increasing depression severity^34^. By decreasing both CAR and morning HR, the Jena Safety Anchor appears to support improved autonomic and neuroendocrine functioning.

These findings suggest that practicing the Jena Safety Anchor may help individuals start their day from a more relaxed, regulated physiological state. This makes it a promising tool not only for coping with isolated stressful events, but also for enhancing stress resilience and overall well-being in everyday life.

## Supplementary Information

Supplementary Information 1.

Supplementary Information 2.

## Data Availability

The datasets generated and analyzed during the current study are available in the Zenodo repository https://zenodo.org/records/17487301.
