# Supplementary figures and images for: Hypnotic safety suggestions reduce cortisol awakening response and morning heart rate in daily life
Source: Sci Rep. 2026 May 8;16:14675. doi: 10.1038/s41598-026-52081-x (PMC13156272; doi:10.1038/s41598-026-52081-x)

# Lower CAR during the week after hypnosis session in hypnosis group

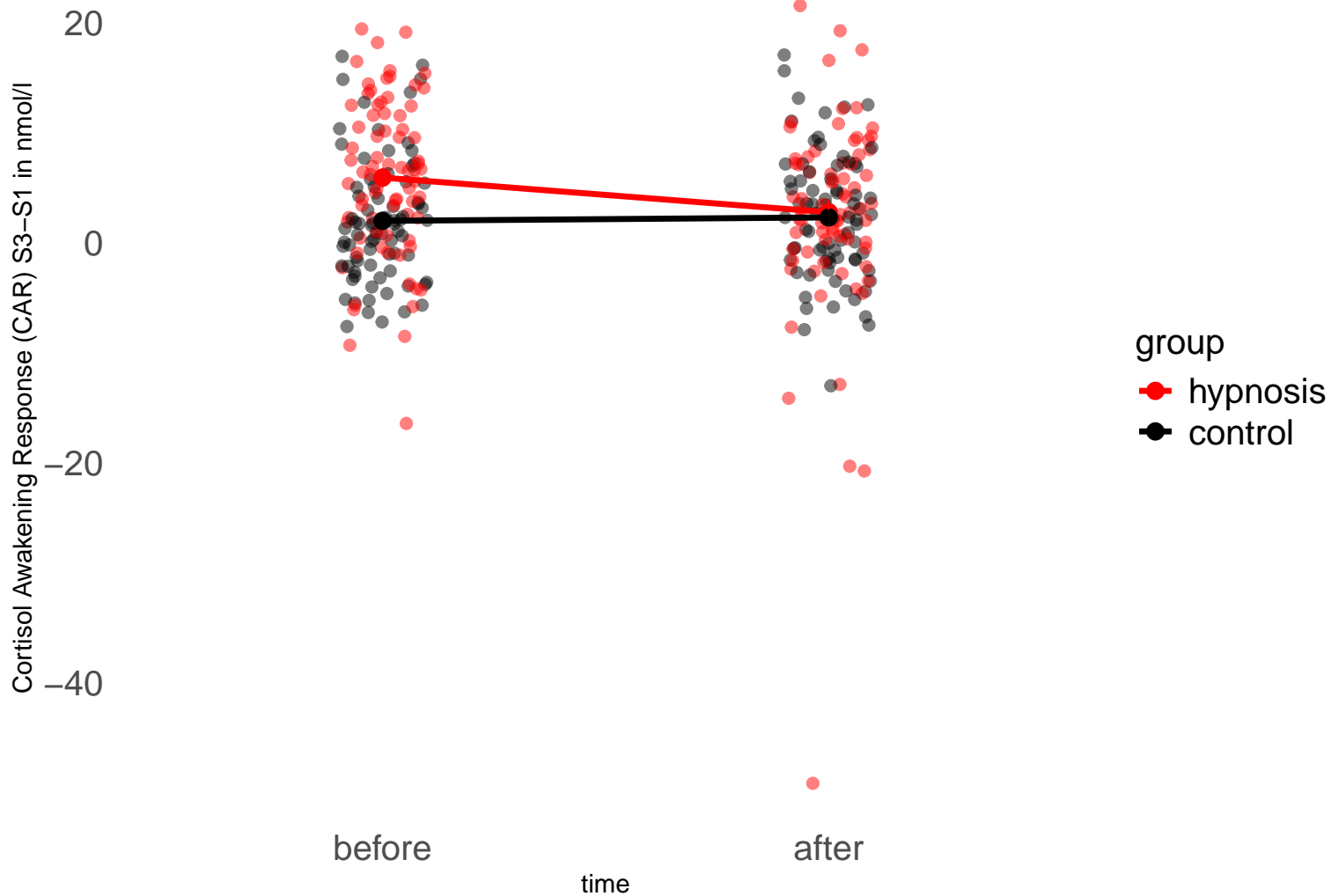

Supplement: Supplementary file 1 — Supplementary Information 1. [file 41598_2026_52081_MOESM1_ESM.pdf]

# Lower morning heart rate after hypnosis session in hypnosis group

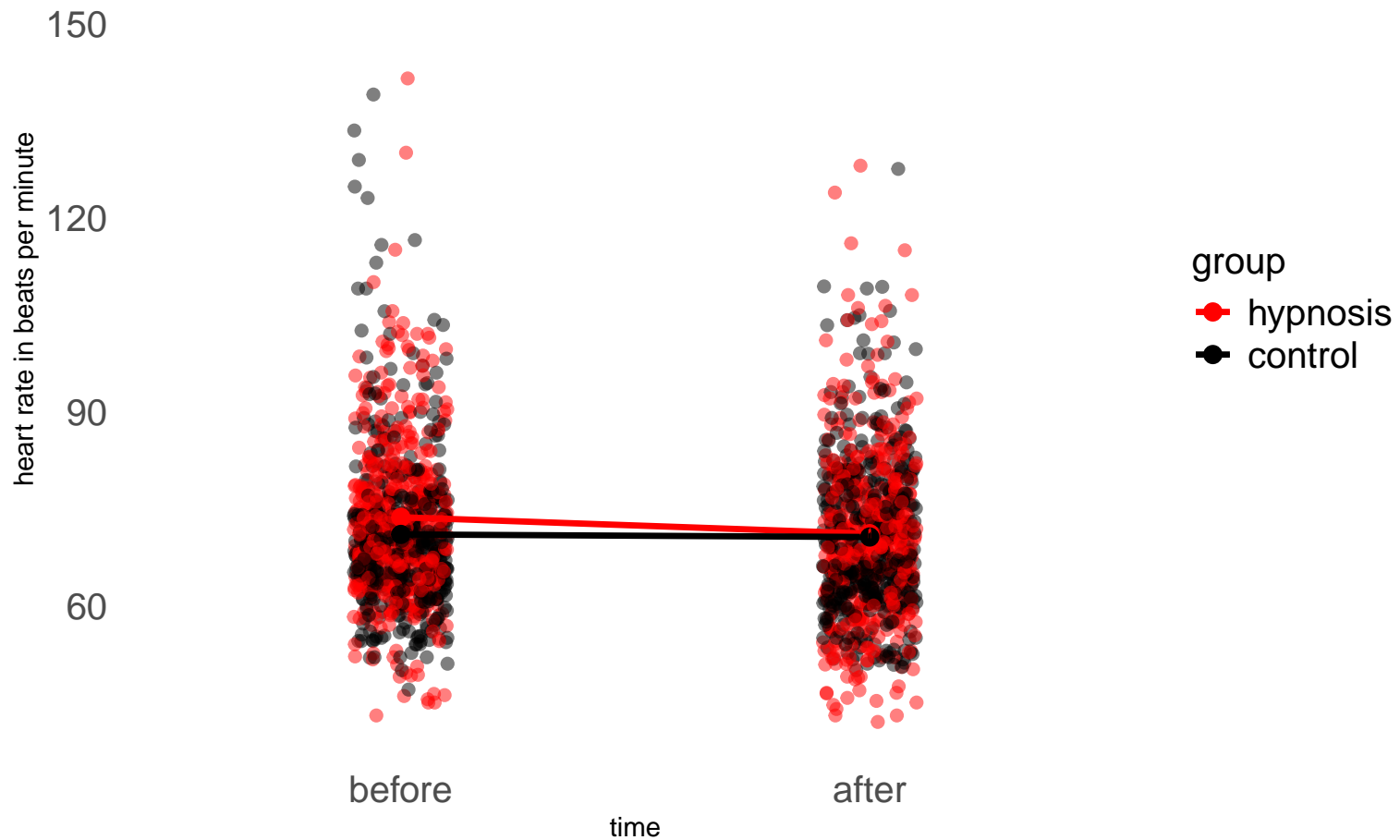

Supplement: Supplementary file 2 — Supplementary Information 2. [file 41598_2026_52081_MOESM2_ESM.pdf]
